# Mystery of Retinal Vein Occlusion: Vasoactivity of the Vein and Possible Involvement of Endothelin-1

**DOI:** 10.1155/2017/4816527

**Published:** 2017-08-20

**Authors:** Teruyo Kida

**Affiliations:** Department of Ophthalmology, Osaka Medical College, Takatsuki, Japan

## Abstract

Retinal vein occlusion (RVO) is a common vascular disease of retina; however, the pathomechanism leading to RVO is not yet clear. In general, increasing age, hypertension, arteriosclerosis, diabetes mellitus, dyslipidemia, cardiovascular disorder, and cerebral stroke are systemic risk factors of RVO. However, RVO often occur in the unilateral eye and sometimes develop in young subjects who have no arteriosclerosis. In addition, RVO show different variations on the degrees of severity; some RVO are resolved without any treatment and others develop vision-threatening complications such as macular edema, combined retinal artery occlusion, vitreous hemorrhage, and glaucoma. Clinical conditions leading to RVO are still open to question. In this review, we discuss how to treat RVO in practice by presenting some RVO cases. We also deliver possible pathomechanisms of RVO through our clinical experience and animal experiments.

## 1. Introduction

Retinal vein occlusion (RVO) is common vascular disease of retina. Some well-known studies in large populations indicate that RVO is the second most frequent retinal vascular disease behind only diabetic retinopathy and that RVO is the fifth leading cause of blindness [1–4]; however, the pathomechanism leading to RVO is not yet clear. In general, RVO can be classified into branch retinal vein occlusion (BRVO) and central retinal vein occlusion (CRVO), depending on the affected vein lesion. Both RVO commonly occur unilaterally and show different variations on the degrees of severity. Some RVO are resolved without any treatment and others develop the vision-threatening complications [4–6], such as macular edema, combined retinal artery occlusion [7, 8], vitreous hemorrhage [9], and glaucoma [10, 11].

Despite taking numerous clinical courses of RVO, these complications are decreasing due to the development of various therapeutic approaches in addition to early alert and treatment by internal medicine doctors for cardiovascular diseases like systemic hypertension and dyslipidemia, which are well-known risk factors of RVO [6, 12–14]. Vein congestion can lead to local hypoxia, thereby increasing vascular endothelial growth factor (VEGF) [15]. Anti-VEGF therapy is the standard treatment for RVO-related macular edema at present, and it is effective in achieving a relatively rapid resolution of macular edema in most RVO patients [16–19]. In addition, it has been reported that not only VEGF but also inflammatory cytokines are associated with RVO-related macular edema [20–22].

Interestingly, the prevalence of RVO in Japan is higher than in other countries [3, 23–27]. The Hisayama study of the general Japanese population aged 40 years or older found that the prevalence of RVO is higher in Japanese patients than in other Asians or Caucasian individuals [3]. In this situation, repeated injections are often required due to a recurrence of macular edema, and the number of injections continues to increase. Thus, it is imperative that we gain a much better understanding of the clinical condition of each patient with RVO and elucidate the undetermined mechanism of RVO.

Many questions remain regarding the clinical condition leading to RVO even though anti-VEGF therapy can save many RVO patients experiencing deteriorated vision. Why does it often occur unilaterally? Does the mechanical “obstruction” or “compression” of the venous lumen induce RVO? Does the affected vein always become thrombosed in the eye with RVO? If we assume a thrombus exists in the affected vein, why is the blood flow not completely disturbed in fluorescein angiography? Are systemic venous thromboembolic (VTE) diseases, such as deep venous thrombosis and pulmonary embolism, not always associated with RVO? Why is BRVO sometimes resolved by itself? Why do young patients sometimes develop RVO without dyslipidemia? Why is the photoreceptor located at the outer retina impaired by the occlusion of the retinal vein located at the inner retina in patients with RVO-related macular edema? Like anti-VEGF therapy, repeated injections of anti-VEGF agent are generally admitted if patients prefer, but can the disorder of the foveal photoreceptor layer by RVO be recovered with improved visual acuity? These questions and others remain unanswered.

In this review, we discuss how to treat patients in practice by presenting some RVO cases. We also deliver possible pathomechanisms of RVO through our clinical experience and animal experiments.

## 2. Clinically Basic Understanding of RVO

### 2.1. Epidemiology of RVO

Retinal vein occlusion (RVO) is the second most frequent retinal vascular disease behind only diabetic retinopathy, and it is the fifth leading cause of blindness [1–4]. Generally, RVO is classified into BRVO and CRVO depending on the affected vein lesion. BRVO was first reported in 1896 [28]. It commonly occurs unilaterally at the arteriovenous crossing. In most cases of BRVO, the artery is located anterior to the vein at the affected arteriovenous crossing [29, 30]. In some cases, the vein is anterior to the artery at the crossing. CRVO is caused by a circulatory disturbance at the trunk of the central retinal vein near the lamina cribrosa. CRVO seems to have been first reported by Coats in 1904, who suggested dividing the clinical conditions of CRVO into two types: those with deteriorated vision and poor prognoses and those with much better vision and prognoses [31].

The prevalence of RVO has been reported by Rogers et al. [32]. They suggested that approximately 16 million people may have the condition, and the age- and sex-standardized prevalence was 5.2 per 1000 for any RVO, 4.42 per 1000 for BRVO, and 0.80 per 1000 for CRVO. There was no difference between the age-standardized prevalence rates in men and women, but the prevalence increases with age [1, 32]. It is known that the major risk factors for RVO include increasing age, systemic hypertension, dyslipidemia, coexisting cardiovascular diseases, and diabetes mellitus [2, 6, 33–37]. Therefore, affected patients are also at higher risk for future cardiovascular events. Interestingly, the risk factor of systemic hypertension is higher in BRVO than in CRVO. In addition, RVO is related to glaucoma [2, 36, 38–40].

### 2.2. Clinical Common Points and Differences between BRVO and CRVO

Both BRVO and CRVO have the following common points: patients with RVO often have systemic hypertension and/or dyslipidemia as a preexisting disease; they show some findings of superficial or deep retinal hemorrhages, cotton wool spots (CWS), venous dilatation, and venous tortuosity in their ocular fundus; they are divided into ischemic and nonischemic [41, 42]; prolonged cases of macular edema lead to poor prognoses. However, while BRVO and CRVO share the commonalities listed above, CRVO is not simply a severe case of BRVO.

There are also significant differences between BRVO and CRVO. CRVO develop neovascular glaucoma, which lead to the risk of blindness [43]. In cases of severe venous obstruction, arterial perfusion is delayed and can be accompanied by artery occlusion [44]. CRVO cases are induced more by variables other than systemic hypertension and arteriosclerosis, unlike BRVO [45]. In addition, natural history is different between BRVO and CRVO. The Branch Vein Occlusion Study showed that visual acuity improved in 40% of eyes with BRVO after 3 years, 34% with visual acuity beyond 20/40 without any treatment [46]. Hayreh and Zimmerman [28] stated that, overall, visual acuity improved or remained stable in about 80% of eyes with BRVO. Rogers et al. [4] reported the natural history of BRVO that visual acuity is generally improved without intervention, although clinically significant improvement beyond 20/40 was uncommon. CRVO differs significantly from BRVO when it comes to its natural history. The Central Vein Occlusion Study found that patients who had poor visual acuity at the first visit (<20/200) had an 80% chance of having a visual acuity less than 20/200 at their final visit, whether perfused or nonperfused initially [41]. In addition, 15% of CRVO eyes with perfusion converted to ischemia in the first 4 months of follow-up, which jumped to 34% after 3 years [41, 47]. Final visual acuity, upon resolution of macular edema, was 20/100 or better in only 12% with ischemic CRVO eyes [48]. McIntosh et al. [49] argued that untreated eyes with CRVO generally have poor visual acuity, which declined further over time. One-quarter of eyes with nonischemic CRVO convert to ischemic CRVO. Thus, early intervention is needed in CRVO, not only ischemic but also nonischemic, to improve vision prognosis.

## 3. Treatments for RVO in Practice

Recently, various therapeutic approaches have been developed, such as anti-VEGF therapy and the intravitreal implant of steroids [50]. For RVO-related macular edema, intravitreal injection of anti-VEGF agent, sub-Tenon's injection of triamcinolone acetonide, grid laser photocoagulation, and vitrectomy are available. For RVO with neovascularization, retinal photocoagulation is effective. In young patients with CRVO, there is a possible involvement of inflammation, and prednisolone is sometimes administered internally [51, 52]. Overall, anti-VEGF therapy seems to be the standard treatment for RVO-related macular edema in recent literatures. Visual acuity is improved in most cases, and there are few complications. It is known that the RVO-related macular edema responds well to intravitreal anti-VEGF because the predominant cause of macular edema is the high levels of VEGF [16, 53]. However, some RVO cases such as having microaneurysms and chronic inflammation do not respond to anti-VEGF agents and repeated injections are needed in chronic state. The high cost of VEGF inhibitors is a significant problem [54]. Thus, it would be efficacious to treat macular edema by other techniques or drugs. To do that, it is necessary to discover the pathomechanisms of RVO.

**Figure 1:**
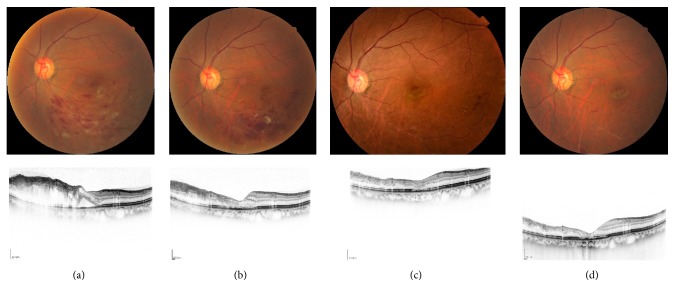
A case of 61-year-old female, BRVO with macular edema. (a) Pretreatment (February, 2014) and (b) one month, (c) six months, and (d) 3 years after intravitreal injection of ranibizumab. Macular edema was decreased and the visual acuity was dramatically improved from 20/50 to 20/20 3 years after the treatment. There has been no recurrence to date.

**Figure 2:**
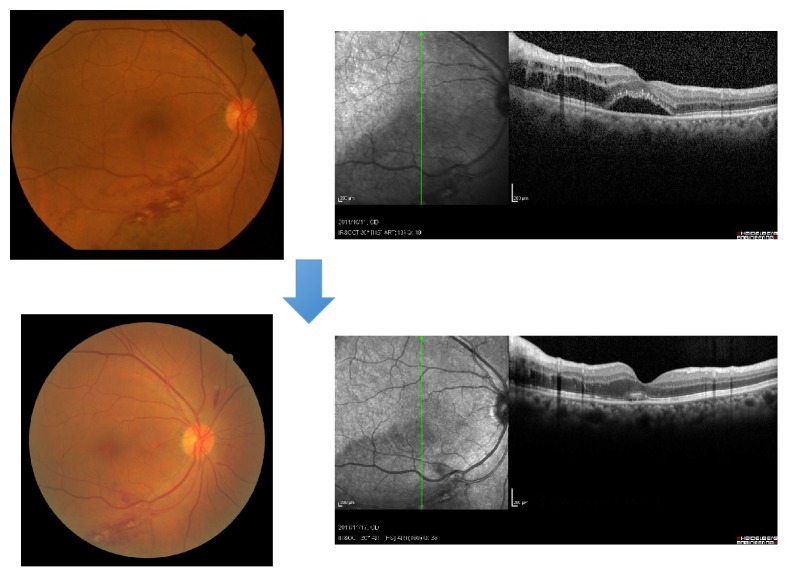
A case of 71-year-old man, BRVO with macular edema before and after treatment of systemic hypertension. His blood pressure was 165/87 mmHg at the first visit. Macular edema was resolved with greatly improved VA from 20/50 to 20/20 1 month after treatment of angiotensin receptor blocker. Anti-VEGF agent was not needed.

**Figure 3:**
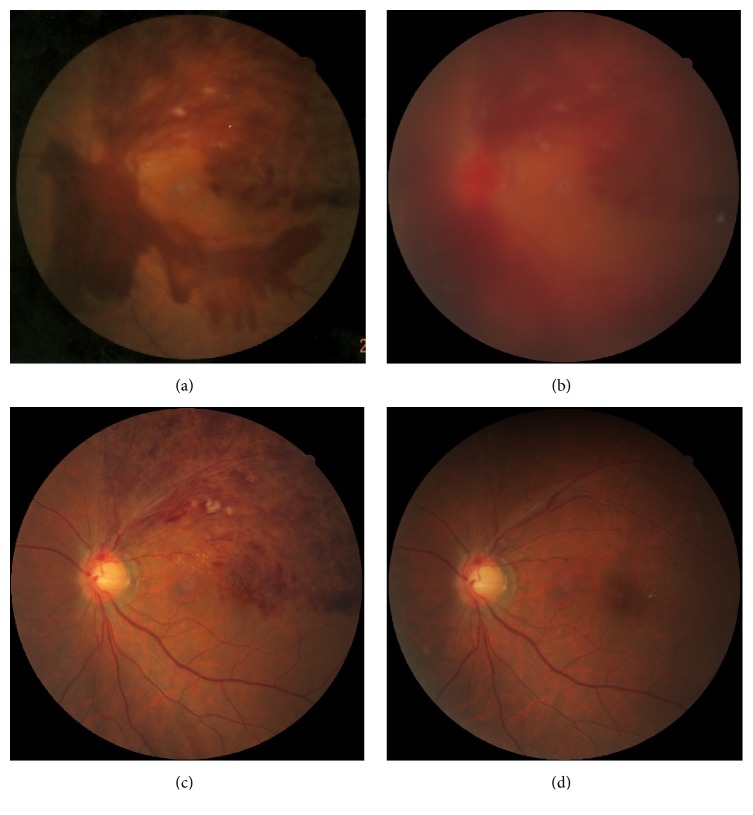
A case of 47-year-old man, preretinal hemorrhage and vitreous hemorrhage (VH) with BRVO. His fundus photos (a) at primary care doctor's clinic (February 1, 2012) and (b) the next day at the first visit. Vitrectomy for VH was planned, but VH was disappeared after one week (c). Laser photocoagulation for large nonperfusion area with neovascularization was conducted. His visual acuity was improved to 20/20 and maintained without recurrence to date (d).

**Figure 4:**
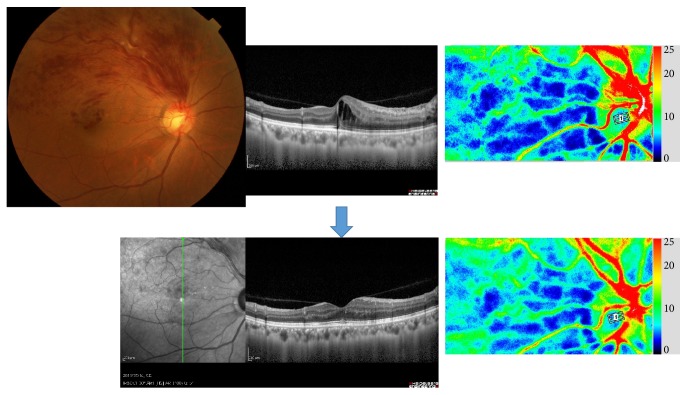
A case of 50-year-old female, BRVO with macular edema. Upper: her visual acuity was 20/25 with macular edema at the first visit. Lower: laser photocoagulation for large nonperfusion area was performed. Her macular edema was resolved with her improved visual acuity of 20/20. We also measured chorioretinal blood flow by laser speckle flowgraphy. Chorioretinal blood flow was not decreased different from a case of anti-VEGF therapy.

**Figure 5:**
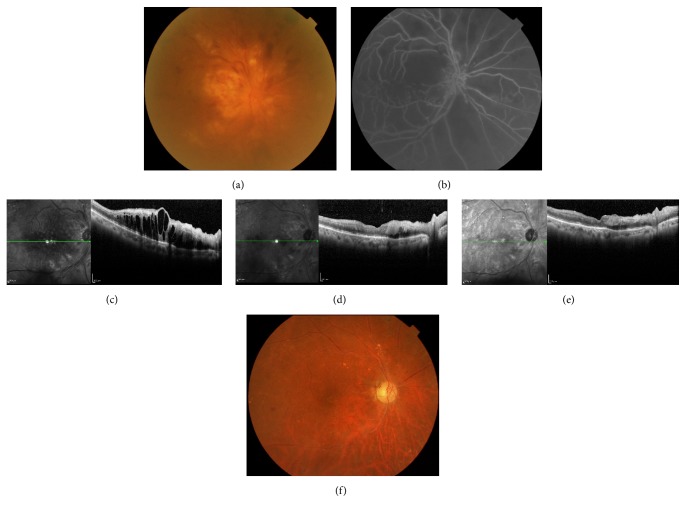
A case of 76-year-old man, ischemic CRVO with cataract. (a) His fundus photo and (b) FA at the first visit. His visual acuity was hand motion. Cataract surgery was performed and then his eye was treated with intravitreal injection of ranibizumab. OCT imaging at (c) pretreatment and (d) one week and (e) one month after anti-VEGF therapy. (f) Fundus photo one year after the treatment. His visual acuity was 20/1000.

In this article, some clinical cases that we experienced are introduced and information on treatment is found in Figures 1–5. In short, at the first visit, we clerk every patient with RVO and measure his or her blood pressure. If the patient's blood pressure is high, we recommend they see an internal doctor to recheck the blood pressure and to run labs including cholesterol, triglycerides, and blood glucose, among others. If the patient's RVO develops macular edema with decreased vision, we consider our earlier intervention of anti-VEGF therapy at the initial visit, especially for some patients who drive a car daily, to improve their visual acuity to be able to drive safely. As a macular finding, foveal subretinal hemorrhage is not uncommon in RVO and may cause subsequent damage to the foveal photoreceptor layer [55, 56]. To avoid the poor visual function, early treatment should be started for foveal subretinal hemorrhage. Subretinal hemorrhage encompasses the atrophy of retinal pigment epithelium (RPE) and fibrosis. Anti-VEGF therapy is also effective for foveal subretinal hemorrhage in RVO.

## 4. RVO and Ocular Circulation

In patients with RVO, retinal circulation is delayed. It is also important to evaluate the degree and area of blood flow disturbance by using fluorescein angiography (FA) and/or optical coherence tomography (OCT) angiography.

There were many efforts made by researchers and ophthalmologists to evaluate the changes in fundus circulation using FA, indocyanine green angiography (IA), laser Doppler velocimetry, laser speckle flowgraphy, and the Oxymap™ in RVO for years before the developed OCT angiography [57–78]. It is important to consider the perfusion status in each patient with RVO. However, it remains unclear why suppression of VEGF in the vitreous by anti-VEGF agents is so effective for macular edema. Thus, we need to detect the changes in chorioretinal circulation and optic nerve head blood flow before and after treatment. To do this, we provide some cases measured by laser speckle flowgraphy before and one month after intravitreal injection of an anti-VEGF drug.

**Figure 6:**
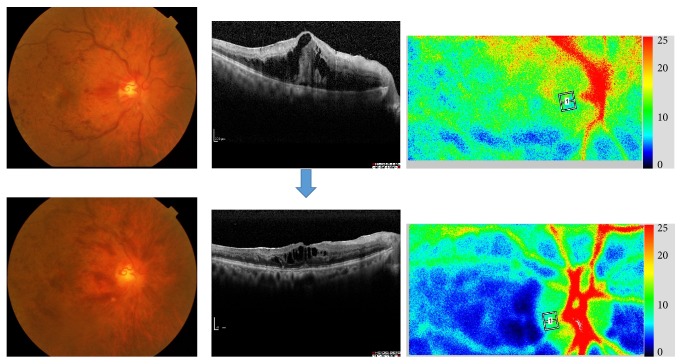
A case of 71-year-old man, CRVO with macular edema. Upper: pretreatment (August, 2011); lower: one month after intravitreal injection of bevacizumab (IVB). Macular edema was decreased and his visual acuity was changed from 20/200 to 20/100. We also measured optic nerve head and chorioretinal blood flow by laser speckle flowgraphy. Blood flow in optic nerve head was not significantly changed; however, chorioretinal blood flow was decreased one month after anti-VEGF therapy.

**Figure 7:**
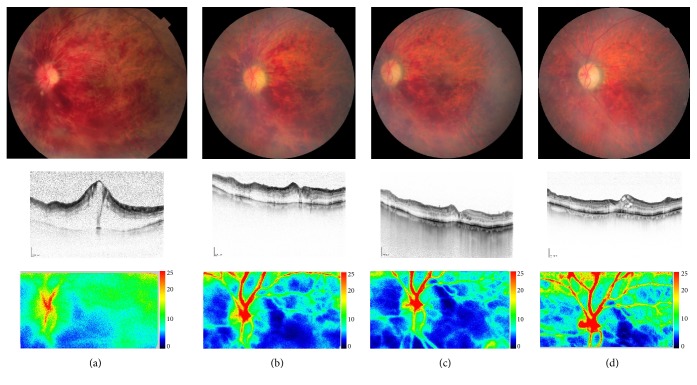
A case of 84-year-old female with CRVO. Her ocular fundus with macular OCTs and imaging of laser speckle flowgraphy at the initial visit (a) and two weeks (b), one month (c), and 3 months after intravitreal injection of ranibizumab (d). Her visual acuity was 20/400, 20/200, 20/200, and 20/100, respectively. Chorioretinal blood flow was decreased two weeks and one month after anti-VEGF therapy and then increased 3 months after the injection.

**Figure 8:**
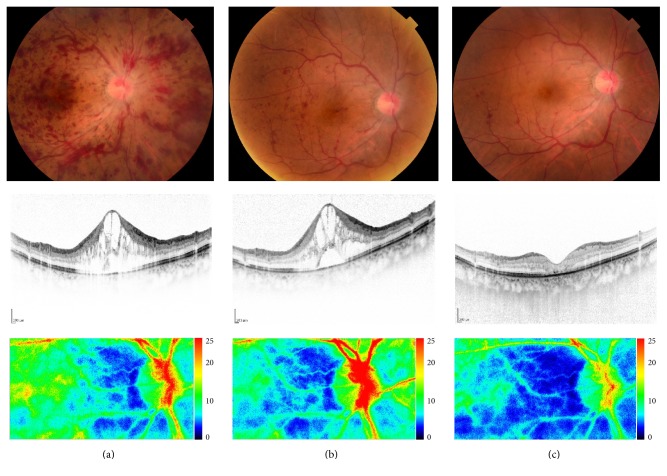
A case of 61-year-old man, CRVO with macular edema (naïve case). His ocular fundus with macular OCTs and imaging of laser speckle flowgraphy at the initial visit (a), after one month of intravitreal injection of aflibercept (b), and after three times of injection (c). Macular edema was not resolved with single injection and his visual acuity was unchanged of 20/100. After three times of anti-VEGF therapy, the macular edema was resolved with visual acuity of 20/60. Chorioretinal blood flow was unchanged one month after anti-VEGF therapy but decreased after three times of anti-VEGF therapy with improved vision of 20/60.

Optic nerve head ocular blood flow did not significantly change before and after the injection of the anti-VEGF agent, but chorioretinal circulation was decreased transiently in most treatment-effective cases of macular edema with RVO (Figures 6 and 7). It is well-known that VEGF upregulates endothelial nitric oxide synthase (eNOS), which in turn synthesizes nitric oxide (NO) [79, 80]. As a result, the blood flow is increased by vasodilation through NO, a vasodilator from vascular endothelium. In our clinical experience, the rate of decrease in chorioretinal tissue circulation following the use of an anti-VEGF drug seems to be case dependent. Interestingly, chorioretinal blood flow did not decrease after treatment in naïve cases of macular edema, which are rarely seen in RVO (Figure 8).

**Figure 9:**
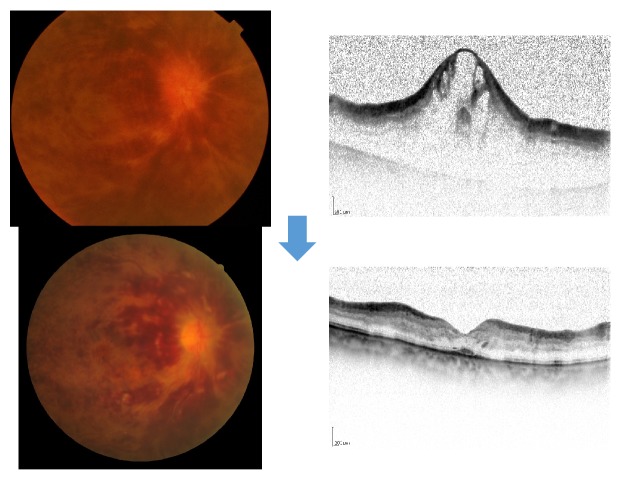
A case of 81-year-old female, ischemic CRVO with macular edema. Cotton wool spots were increased one month after intravitreal injection of aflibercept. However, macular edema was dramatically decreased without change of her visual acuity of 20/200.

**Figure 10:**
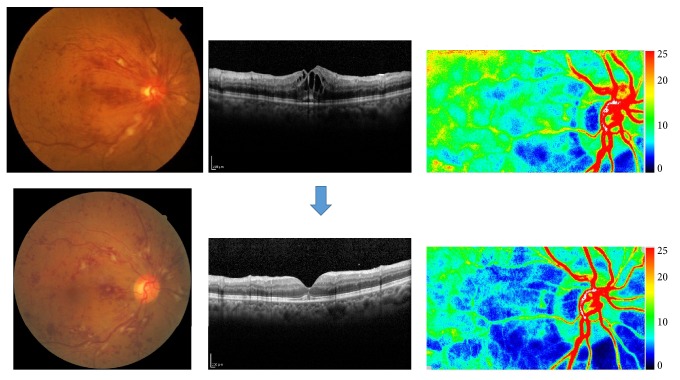
A case of 65-year-old man, CRVO with macular edema. Cotton wool spots were increased one month after intravitreal injection of aflibercept. However, macular edema was dramatically decreased, and his visual acuity was improved from 20/60 to 20/20.

During our clinical researches for ocular blood flow in patients with RVO [37, 81], we encountered a transient increase in retinal cotton wool spots (CWS) following anti-VEGF therapy for the treatment of macular edema secondary to CRVO (Figures 9 and 10). We reported a variety of ocular fundus photos in patients with CRVO [82]. CWS are accumulations of cytoid bodies formed through overproliferation and degeneration of axoplasmic organelles, and they are recognized as signs of acute ischemia within the nerve fiber layer [57, 83]. If too much suppression of VEGF by an anti-VEGF agent occurs in a patient with RVO-related macular edema, it might induce too much decrease of chorioretinal blood flow, and there are some reports of ischemic change after anti-VEGF therapy [84–90]. VEGF is known to stimulate the production of NO, which is an endothelium-derived relaxing factor, and then increases the blood flow. Anti-VEGF agents such as bevacizumab have a vasoconstrictor effect [87], and the animal experiment showed that bevacizumab immune complexes activate platelets and induce thrombosis in mice [91, 92]. We found a transient increase of CWS after anti-VEGF therapy in patients with CRVO, but macular edema, retinal hemorrhage, and visual acuity were improved in almost every case [82].

Recently, the technology of OCT imaging has progressed rapidly, and OCT angiography is a popular tool for evaluating the pathology of RVO. Some papers found out that OCT angiography can visualize microvascular abnormalities equally well or better than FA [93, 94], and the deep capillary plexus appears to be more severely affected than the superficial capillary plexus in RVO [95]. This finding might lead to the discovery of the pathological condition of abnormal blood flow in RVO.

## 5. Possible Pathomechanism of RVO

The pathomechanism leading to RVO is not yet clear. BRVO commonly occurs at the arteriovenous crossing. It is assumed that the diseased artery mechanically compresses the vein. However, it has been reported that the retinal vein seems to run deep under the artery at the crossing in eyes with arterial overcrossing, and the venous lumen often appears to be preserved, even at the arteriovenous crossing, as observed by thin sectioning with optical coherence tomography (OCT) in patients with acute BRVO [30, 96]. In addition, it is possible that the vein locally constricts actively due to an altered biochemical environment.

The tortuosity of the vein is one clinical finding in CRVO. Muraoka et al. [97] reported that OCT can be used to visualize anteroposterior venous tortuosity and associated structural changes to the retinal parenchyma. This indicates that the vein itself has vasoactivity, and this phenomenon is associated with the pathomechanism of CRVO. Reviewing papers regarding RVO and vasoactivity, a brief report by Catier et al. 14 years ago found that a patient with vasospasms during impending CRVO was found by taking indocyanine green videoangiography [63]. In addition, Paques et al. [98] found venous nicking without arteriovenous contact by adaptive optics imaging in 2015. Recently, Yu et al. [99] suggested local modulation of retinal vein tone by using isolated perfused porcine retinal vein. CRVO is found at the trunk of the central retinal vein near the lamina cribrosa. Kang et al. [100] documented the morphometric features of arterial and venous endothelia in the different laminar regions of normal human optic nerve heads. They found an arterial-like appearance of venous endothelium in the posterior lamina cribrosa, where pressure gradient forces are predicted to be greatest and the luminal diameter of the central retinal vein is known to be narrowest, which implicates this as a site of altered hemodynamic stress. These findings by above authors are potential ways to visualize vasoactivity of the vein, and it is important to find out the pathomechanism of CRVO.

In addition, these interesting new findings may be able to act, leading to CRVO and normal tension glaucoma (NTG). The pathomechanism of NTG is also unknown. Because of this, we retrospectively investigated 234 Japanese CRVO patients over 5 years, with follow-ups of more than 12 months, and evaluated the prevalence of glaucoma [101]. Of the 234 CRVO patients, 18 (7.7%) were diagnosed with 10 NTG or 8 primary open-angle glaucoma cases (POAG). The proportion of NTG with systemic hypertension was low. Interestingly, intraocular pressure (IOP) of NTG patients was significantly elevated at the initial CRVO evaluation, even in the presence of antiglaucoma drugs. It is already known that increased retinal venous pressure occurs in RVO [102], and, among the patients with POAG, spontaneous venous pulsation was less common in patients with low IOP at all stages of disease [103].

**Figure 11:**
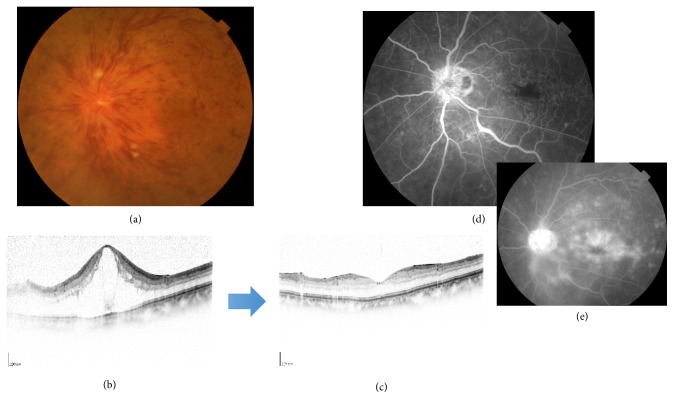
A case of 87-year-old man, CRVO with macular edema. His macular edema was dramatically decreased after intravitreal injection of aflibercept ((b) and (c)). His visual acuity was improved only a little from 20/200 to 20/100. His FA showed the enlargement of foveal avascular zone (FAZ) in early phase (d). His OCT finding (c) after the treatment looked normal macular shape, but the photo in the late phase of FA showed fluorescein leak in the macula (e).

Going back now to RVO and VEGF, the congestion of the vein in RVO can lead to local hypoxia, thereby increasing VEGF, which in turn contributes to macular edema [104, 105]. Anti-VEGF therapy leads to the rapid reduction of macular edema and an improvement in visual function [106]. However, anti-VEGF therapy does not improve visual acuity in every patient with RVO, although a regression in macular edema is present after performing the therapy (Figure 11). This indicates that factors other than VEGF are potentially involved as well.

**Figure 12:**
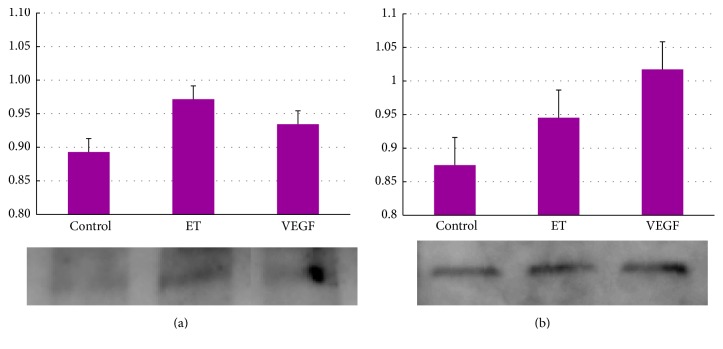
Western blot analyses of HIF-1 and VEGF in rat cultured retinal Müller cells, respectively. The exposure to ET-1 and VEGF increased expressions of HIF-1 (a) and VEGF (b).

One possible candidate is endothelin. Endothelin-1 (ET-1) is a potent vasoconstrictor [107]. It regulates the blood-retinal barrier, stimulates the growth and migration of cells, and regulates axoplasmic transport. It is essential for the maintenance of cardiovascular homeostasis [108]. While ET-1 is mainly produced by vascular endothelial cells under physiological conditions, it can be produced by any other cell under pathological conditions, such as hypoxia or inflammation [109, 110]. The increase of VEGF points to a local hypoxia [111, 112]. Hypoxic cells oxidize less of the hypoxia-inducible factor-1 alpha (HIF). The nonoxidized HIF is a transcription factor that upregulates the transcription of many genes. As a result, VEGF, ET-1, or erythropoietin and other molecules are overexpressed (Figure 12).

**Figure 13:**
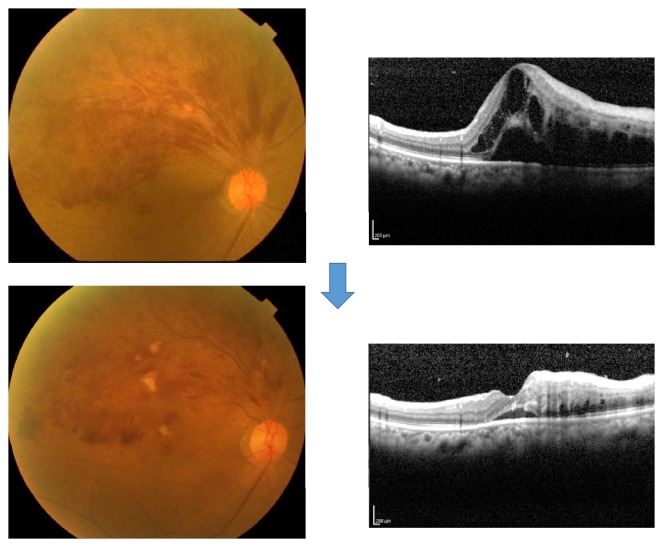
A case of 68-year-old female, BRVO with macular edema. Upper: pretreatment; lower: one month after intravitreal injection of bevacizumab (IVB). Macular edema was dramatically decreased and the visual acuity was improved from 2/20 to 8/20 one month after IVB. Plasma ET-1 level was decreased from 1.0567 to 0.568 pg/mL.

With this in mind, we conducted a prospective study to evaluate changes in ET-1 following an injection of intravitreal bevacizumab (IVB), which is the anti-VEGF agent with the longest serum half-life [113], to determine its effect on BRVO-related macular edema [114]. We found that plasma ET-1 levels dropped and macular edema was resolved after anti-VEGF therapy in most patients (Figure 13). However, some patients showed an increase in the plasma ET-1 level with slightly decreased visual acuity. It means that anti-VEGF agents reduce intraocular VEGF levels and blood ET-1 levels.

The overexpression of VEGF hints at a simultaneous overexpression of ET-1, and, most probably, both VEGF and ET-1 are involved in the pathogenesis of RVO-related macular edema. VEGF is known to stimulate the production of NO, which is an endothelium-derived relaxing factor, and then increases the blood flow, as described in RVO and Ocular Circulation. If the relation among VEGF, NO, and ET-1 became unbalanced in the retina, RVO might occur. It is known that a reduction of VEGF normally also leads to a reduction in ET-1 [115, 116]. However, the reactive increase in the ET-1 level after treatment with the anti-VEGF agent raises the question of whether or not the combination of an anti-VEGF therapy with an anti-ET-1 treatment could improve visual prognosis in these patients. In most RVO patients, anti-ET-1 treatment might not be necessary because anti-VEGF agents can neutralize their intraocular VEGF levels.

**Figure 14:**
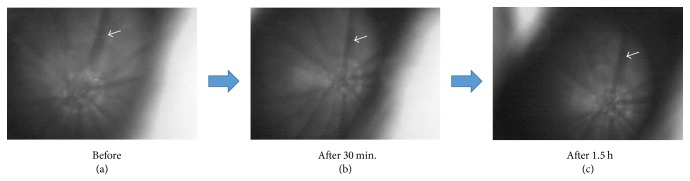
Changes of vessels in ocular fundus following intravenous injection of ET-1 in rats. (a) Pretreatment and (b) 30 minutes and (c) 1.5 hours after the injection. The vasospasm (arrow) was observed 30 minutes after the injection.

**Figure 15:**
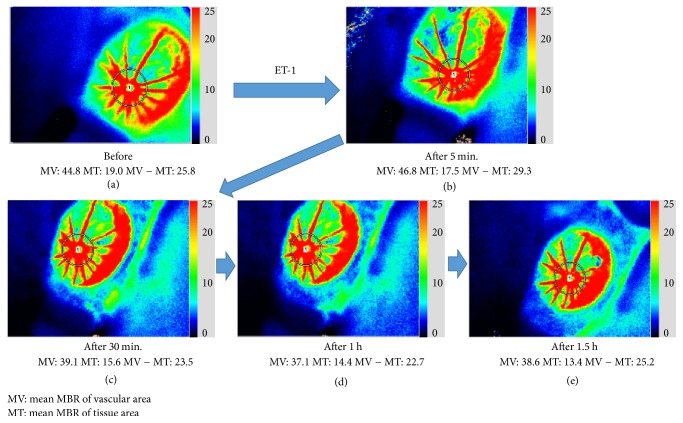
Measurement of retinal blood flow by laser speckle flowgraphy before and after intravenous injection of ET-1 in rats. (a) Pretreatment, (b) 5 minutes, (c) 30 minutes, (d) 1 hour, and (e) 1.5 hours after the injection. Retinal blood flow (MV-MT) was slightly increased 5 minutes after the injection (b) and then decreased at 30 minutes (c) and returned to the initial level 1.5 hours after the injection (e).

In an RVO experiment, ET-1 blockers were found to improve retinal circulation [117, 118]. In patients with RVO, calcium channel blockers for systemic hypertension and reducing vasospasm were found to reduce retinal venous pressure [119, 120]. This partly reduces the effect of ET-1. Finally, Figures 14 and 15 introduce our simple animal experiment of retinal vasospasm by ET-1. Changes of vessels in ocular fundus following intravenous injection of ET-1 in rats were investigated. The vasospasm in one of retinal vessels was observed 30 minutes after the injection of ET-1 (Figure 14). In addition, retinal blood flow by laser speckle flowgraphy before and after intravenous injection of ET-1 in rats was measured at the same time. Interestingly, retinal blood flow was slightly increased 5 minutes after the injection and then decreased at 30 minutes and returned to the initial level 1.5 hours after the injection (Figure 15). This finding might induce the pathological condition of RVO.

## 6. Conclusions

Retinal vein occlusion (RVO) is common retinal vascular disease. Anti-VEGF therapy is now the standard treatment and is effective for RVO-related macular edema in most cases. However, RVO have a variety of clinical conditions and its pathomechanism remains unknown. We provided some ways of distinguishing RVO patients in practice and introduced the vasoactivity of the vein itself and the possible involvement of vasoconstrictors such as ET-1 for the pathogenesis of RVO through our clinical studies, some animal experiments, and recent literatures. Treatment of systemic hypertension and correction of dyslipidemia by improving patients' diets are still important to avoid developing both RVO and cardiovascular diseases [13]. Further investigation is needed to reach the goal of improved 20/20 vision without recurrence in every patient with RVO.
